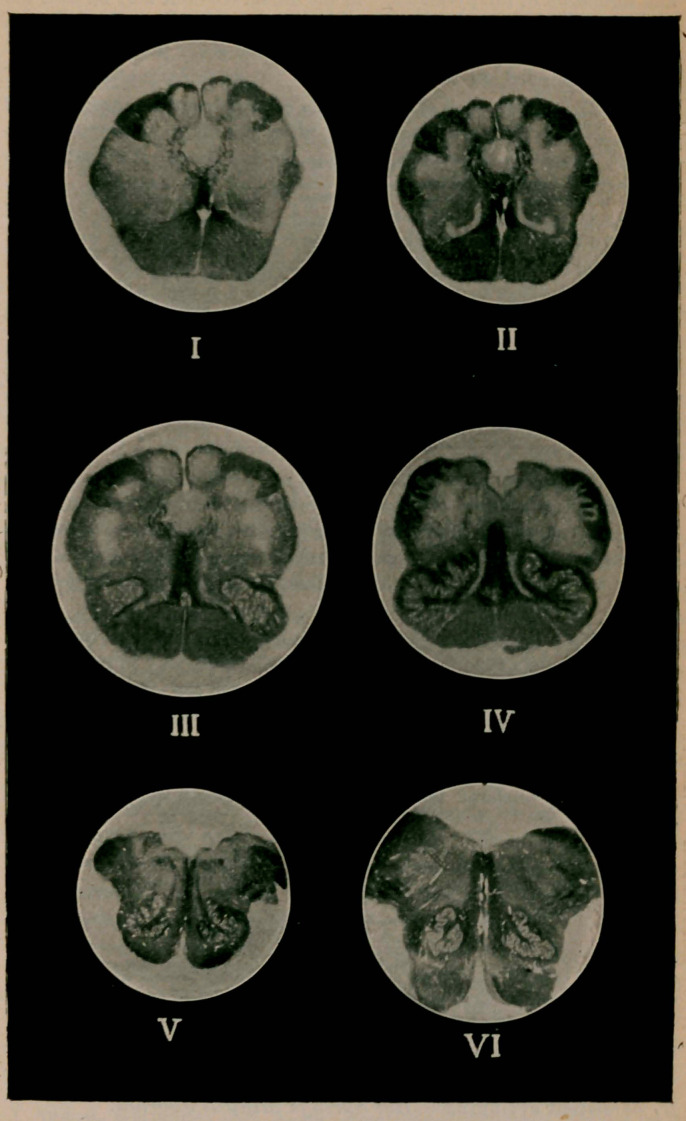# The Histological Conformation of the Medulla1Illustrated by a series of six slides, to which was awarded the cash prize of the American Microscopical Society, held at Madison, Wis., Aug. 19-21, 1893.

**Published:** 1895-08

**Authors:** William C. Krauss

**Affiliations:** Professor of nervous diseases in the Medical Department of Niagara University, Buffalo, N. Y.


### THE HISTOLOGICAL CONFORMATION OF THE MEDULLA.[1]

By WILLIAM C. KRAUSS, M. D., F. R. M. S.,

Professor of nervous diseases in the Medical Department of Niagara University, Buffalo, N. Y.

THE study of the transition of the cord to the brain is perhaps the most difficult task in the anatomy and histology of the nervous system. To trace the origin and direction of nerve bundles, the appearance of new masses of gray matter, and the coalition of different tracts requires much time, excellent specimens, correct drawings and careful explanations. The discovery of the Weigert method of staining and the Pal modification has proved of great benefit in following these complicated changes, and permitted the investigator to trace the course of the different tracts with much satisfaction and success.

The internal configuration of the spinal cord from the third or fourth lumbar segment to the second or third cervical is practically the same. Slight changes occur characteristic of the lumbar, thoracic and cervical regions, such as the development of the dorsal cornua, or lateral cornua, or of the ventral cornua; but these are of minor importance when compared to the changes occurring between the first and second cervical segment at the beginning of the pons.

The spinal cord within the limits referred to consists of a cylindrical mass of nerve elements held together by neuroglia cells and encased in appropriate membranes, lending support, protection and nutrition. The white matter, composed of medullated nerve fibers, is arranged peripherally, while the gray matter containing the ganglion cells is situated in the interior. The gray matter consists of two crescentic masses, their convex surfaces facing and connected together by a bridge of gray matter, the commissure; the whole resembling very much the letter H. The gray matter is divided arbitrarily into dorsal, lateral and ventral cornua, and anterior gray commissure inclosing the spinal canal. From the dorsal and ventral cornua bundles of nerve fibers pass through the white matter to the periphery of the cord, forming the dorsal and ventral spinal roots.

The white matter is divided into two hemispheres by the dorsal and ventral fissures, and each hemisphere is separated into dorsal, lateral and ventral columns. These columns are further subdivided as follows: the ventral, into the columns of Türck and ventral columns proper; the lateral, into the pyramidal tracts, cerebellar tracts (Flechsig), and tracts of Gowers; the dorsal, into the columns of Goll, columns of Burdach and Spitzka-Lissauer tracts. These subdivisions are mapped out according to the part they play in conducting nerve impulses to or from the brain centers. The continuation of the cord caudad, is the cauda equina; cephalad, the medulla.

From about the middle of the thoracic region fibers pass from the pyramidal tracts in the lateral columns through the base of the ventral cornua to the ventral columns of the opposite side. This decussation is scarcely recognisable at first, but gradually increases cephalad until the first cervical segment, when the remaining fibers not yet decussated pass to the opposite side in bundles, severing the ventral cornua from the central gray matter and pushing them laterad. The lateral cornua become prominent, and from their cells originate the XI. pair of nerves (spinal accessory), passing through the white matter and emerging at the side of the medulla between the dorsal and ventral roots.

While these changes are going on in the ventral and lateral columns the dorsal columns increase in size, the funiculi graciles (tracts of Goll) and funiculi cuneati (tracts of Burdach) develop, pushing the dorsal cornua before them. The long slender bodies of the dorsal cornua swell, enlarge, become rounded and are connected to the central gray matter by thin pedicles. The substantia gelatinosa Rolandi becomes prominent, and medullated nerve fibers arise adjacent and ectad, passing cephalad as the ascending branch of the trigeminus. The formatio reticularis develops in the lateral columns, appropriating to itself the severed portions of the ventral cornua, except some small aggregations of nerve cells surrounded by patches of gray matter, the most important of which is called nucleus lateralis medius or nucleus ambiguus.

Further cephalad there arise in the funiculi graciles and cuneati, small masses of gray matter containing isolated groups of ganglion cells designated nuclei funiculi graciles and nuclei funiculi cuneati. The decussation of the pyramidal fibers being completed and the pyramids fully developed, other fibers may be observed coming from the former dorsal columns, passing concentrically about the central canal and decussating ventrad of it, then arranging themselves on both sides of the ventral fissure dorsad of the pyramids forming the lemniscus.

The continuance of these fibers, now designated fibræ arcuatæ internæ decussate in the median line, from the central canal to the ventral fissure forming the raphe.

In the lateral regions immediately dorsad of the pyramids small masses of ganglion cells, symmetrically arranged, begin to appear as convoluted intestinal-like masses called the olivary bodies. Generally there are two or three of these bodies present besides the principal ones, and to these have been given the names internal and external accessory olivary processes.

The central canal, at first circular, has become elliptical, and traced cephalad it widens out, rises to the dorsal surface and terminates in the calamus scriptorius, then into the fourth ventricle. In the base of the ventral cornua a group of large multipolar ganglion cells appear, the nucleus of the XII. pair or hypoglossal nerves. The nerve fibers coming from these cells pass ventrad through the formatio reticularis, between the olivary bodies and the pyramids, and emerge exteriorly laterad of the pyramids. In the base of the dorsal cornua, now pushed latero-dorsad and appearing as the gray matter in the floor of the fourth ventricle, appear masses of small ganglionic cells, the nuclei of the X., IX., and VIII. pairs of nerves.

The cerebellar tracts or tracts of Flechsig have joined with other fibers coming from the dorsal and lateral columns and pass cephalad into the cerebellum as the corpora restiforme.

In the floor of the fourth ventricle, close beside the median raphe, a small group of ganglion cells may be observed—the nucleus funiculi teretis. Ventrad of these cells an oval bundle of nerve fibers passing cephalad may be seen, called the fasiculus longitudinal posterior.

The next important change is the appearance of the transverse bundles of fibers forming the pons, and with the appearance of these fibers the medulla ceases its existence.

### EXPLANATION OF PLATE.

Fig. I.—Transection of the medulla at its junction with the cord (first cervical segment), showing the decussation of the pyramidal tracts

I
II
III
IV
V
VI

and formation of the pyramids. In the dorsal columns may be seen the newly-formed funiculi cuneati and graciles, and in the lateral columns the origin of the fibers of the ascending branch of the trigeminus nerve. Magnified two and one-half diameters.

Fig. II.—Transection of the medulla one-half centimeter cephalad of Figure I., showing the decussation of fibers (sensory) coming from the funiculi cuneati and graciles, forming the lemniscus. Laterad of the lemniscus appear the olivary bodies. Magnified two diameters.

Fig. III.—Transection of the medulla one-half centimeter cephalad of Figure II., showing the fibræ arcuate internæ decussating and forming the raphe. The olivary bodies are more developed, and the spinal canal may be seen to gradually ascend to the dorsal surface, where, further cephalad, it terminates in the calamus scriptorius, then into the fourth ventricle. Magnified two and one-half diameters.

Fig. IV.—Transection of the medulla one-half centimeter cephalad of Figure III., showing the calamus scriptorius. The olivary bodies and the accessory olivary bodies are now plainly visible. Magnified two diameters.

Fig. 5.—Transection of the medulla one centimeter caudad of the pons, showing the floor of the fourth ventricle, the development of the restiform bodies, the fibers of the hypoglossal nerves separating the substantia reticularis alba from the substantia reticularis grisea, and the ascending branches of the glosso-pharyngeal nerves. Magnified one and one-fourth diameters.

Fig. VI.—Transection of the medulla one-half centimeter caudad of the pons, showing the fasiculus longitudinalis posterior in the floor of the fourth ventricle and indistinctly the emergence of the fibers of the vagus nerves. Magnified one and three-fourth diameters.

The nuclei of the XII., XI., X., IX., and VIII., pairs of cranial nerves are situated in the medulla embraced by these sections, but on account of their being rendered transparent by the Pal method, cannot be distinguished.

1. Illustrated by a series of six slides, to which was awarded the cash prize of the American Microscopical Society, held at Madison, Wis., Aug. 19-21, 1893.

---

IMPACTED CERUMEN.—To dislodge hard, impacted wax from the ear, Dr. Dundas Grant (London), *Medical Times or Hospital Gazette*, recommends a solution, consisting of fifteen grains of bicarbonate of soda, three drachms of glycerine and distilled water to make an ounce ; to be dropped into the ear, warm, followed by persistent syringing.—*Hot Springs Medical Journal.*

## Figures and Tables

**Figure f1:**